# Identification of cerebrospinal fluid metabolites as biomarkers for neurobrucellosis by liquid chromatography-mass spectrometry approach

**DOI:** 10.1080/21655979.2022.2037954

**Published:** 2022-03-07

**Authors:** Hao Yang, Zhenfei Wang, Shujun Shi, Qin Yu, Meiling Liu, Zhelin Zhang

**Affiliations:** a Department of Radiation Oncology, Inner Mongolia Cancer Hospital & Affiliated People’s Hospital of Inner Mongolia Medical University, Hohhot, China; b Department of Neurology, Affiliated Hospital of Inner Mongolia Medical University, Hohhot, China

**Keywords:** Neurobrucellosis, metabolomics, cerebrospinal fluid, liquid chromatography-mass spectrometry

## Abstract

Neurobrucellosis is the most morbid form in brucellosis disease. Metabolomics is an emerging method which intends to explore the global alterations of various metabolites in samples. We aimed to identify metabolites in cerebrospinal fluid (CSF) as biomarkers that were potentially unique for neurobrucellosis. CSF samples from 25 neurobrucellosis patients and 25 normal controls (uninfected patients with hydrocephalus) were collected for metabolite detection using liquid chromatography-mass spectrometry (LC-MS) approach. Inflammatory cytokines in CSF were measured with Enzyme-linked immunosorbent assay (ELISA). The base peak chromatogram in CSF samples showed that small-molecule metabolites were well separated. Principal Component Analysis (PCA) analysis exhibited the examined samples were arranged in two main clusters in accordance with their group. Projection to Latent Structures Discriminant Analysis (PLS-DA) revealed there was a noticeable separation between neurobrucellosis and normal groups. Orthogonal Partial Least-Squares-Discriminant Analysis (OPLS-DA) could responsibly illuminate the differences between neurobrucellosis and normal controls. Neurobrucellosis showed a total of 155 differentiated metabolites. Prominent potential biomarkers including 30 metabolites were then selected out, regarded as more capable of distinguishing neurobrucellosis. TNF-α and IL-6 in CSF were remarkably increased in neurobrucellosis. We presented the heatmaps and correlation analyses among the identified 30 potential biomarkers. In conclusion, this study showed that CSF metabolomics based on LC-MS could distinguish neurobrucellosis patients from normal controls. Our data offered perspectives for diagnosis and treatment for neurobrucellosis.

## Graphical abstract

**Figure:**
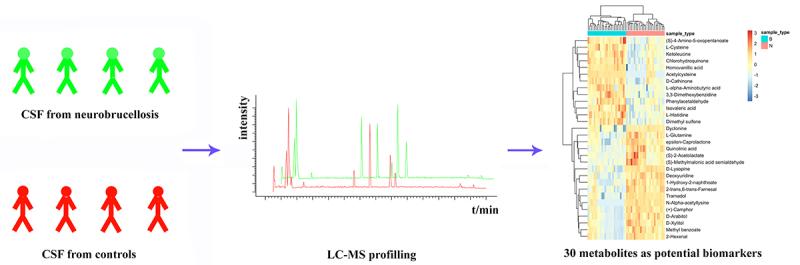

## Introduction

Brucellosis is a weakening febrile disease induced by intracellular Brucella infection which is widely distributed in both animals and humans [1]. Neurobrucellosis is a rare complication of brucellosis involving central nervous system [2]. It usually begins with neurological symptoms, which can escape innate immunity to maintain intracellular survival and reproduction [3]. With neither specific clinical manifestations nor specific cerebrospinal fluid (CSF) image, neurobrucellosis always simulates other infectious neurological diseases, resulting in diagnostic difficulty [4]. Due to the great dilemma of obtaining clinical samples and the risk of infection, research on neurobrucellosis is still limited. It is very essential to find out molecular markers that can quickly diagnose the disease and provide strong evidence for subsequent treatment and prognosis.

Metabolomics is a developing approach for investigating the global alterations of various metabolites in samples [5]. Metabolites are deemed to the most direct manifestation in pathological situations, providing profound understanding for disease phenotype [6]. For instance, novel metabolites found in patients with premature ovarian failure and polycystic ovarian syndrome can display good diagnostic performance and act as effective biomarkers [7]; Tricarboxylic acid cycle and caffeine metabolism have been validated to play critical roles in vascular risk factors-related cognitive damage [8]. Through profiling metabolites in biofluids such as blood and CSF, metabolomics becomes an encouraging and strong strategy to offer precious insights for the etiopathogenesis of neurological disorders [5]. Using metabolomics to explore neurological diseases allows the research on metabolite entities which are small enough to cross blood-brain barrier, probably serving as a reservoir for searching biomarkers [9]. Great advance in targeted metabolomics analysis benefits from the improvement of liquid chromatography-mass spectrometry (LC-MS) device and analytical approach [10]. This technique is currently able to perform accurate targeted detection of numerous metabolites in biological samples [11].

Metabolomics based on LC-MS has been applied in investigating CSF samples in neurological disorders [12,13]. Proteomic analysis reveals that environmental stress induces metabolic adaptation in Brucella abortus, including oxidative phosphorylation, citrate cycle, thiamine metabolism and nitrogen metabolism [14]. Genome-wide core proteome analysis exhibits that 31 proteins are found to be involved in 10 metabolic pathways which are unique to Brucella melitensis [15]. In-depth profiling of CSF metabolome can be performed by chemical isotope labeling LC-MS for identifying some latent molecular markers in experimental ischemic stroke [16]. However, metabolomics study in brucellosis is deficient; besides, CSF metabolites in neurobrucellosis patients are also ambiguous.

We hypothesized that LC-MS research in CSF might present abnormal metabolite models, which have the possibility to function as alternative targets for scrutinizing neurobrucellosis development. In the present study, we aimed to detect potential metabolites in CSF that were distinctively correlated with neurobrucellosis using LC-MS approach. Applying metabolomics to explore neurobrucellosis would offer a systematic method to understand neurobrucellosis pathology and to recognize biomarkers.

## Materials and methods

### Cerebrospinal fluid (CSF) samples from patients *[2,17,18]*

The cases involved patients recruited from the Affiliated Hospital of Inner Mongolia Medical University between 1 June 2019 and 1 June 2020. CSF samples were collected from 25 uninfected patients with hydrocephalus (Control group) and 25 neurobrucellosis patients.

The diagnosis of neurobrucellosis met the following conditions: 1) clinical manifestations in line with the known neurobrucellosis syndrome; 2) typical CSF alterations (pleocytosis and increased protein concentration); 3) positive outcomes in the culture or serological tests of blood, bone marrow or CSF. For control group, the samples of CSF were obtained from patients with venous sinus thrombosis, hydrocephalus, headache (uninfected individuals that confirmed by laboratory tests). CSF samples were collected before treatment. Written informed consents were gained for all participants prior to their involvement in this research. Our study had been approved by ethics committee of the institution and performed in accordance with the Declaration of Helsinki.

### Sample extraction *[18-20]*

All samples were thawed at 4°C. The insufficient specimens were decreased to the equal scale. Each sample (100 μL) was removed into centrifuge tubes (2 mL). Methanol (400 μL, Thermo Fisher, Waltham, MA, USA) was added to each tube and vortexed for 60 s. After centrifuged at 12,000 rpm for 10 min at 4°C, all supernatant from each specimen was removed into another centrifuge tube (2 mL). Specimens were concentrated and dried within vacuum, then they were dissolved by 2-chlorobenzalanine (150 μL, 4 ppm, Aladdin Reagent, Shanghai, China) in 80% methanol solution. 0.22 μm membrane was used to filter the supernatant for obtaining the prepared specimens for LC-MS. For monitoring deviations of the analytical results from the pool mixtures and comparing them to the errors produced by the analytical instrument, each sample (20 μL) was taken as the quality control samples. The rest of the specimens were used for LC-MS measurement.

### Chromatographic condition *[19,20]*

The Thermo Vanquish system (Thermo Fisher) equipped with an ACQUITY UPLC® HSS T3 (1.8 μm, 150 × 2.1 mm, Waters) column was applied for chromatographic separation, maintaining at 40°C.

The temperature at the autosampler was 8°C. With formic acid (0.1%, TCI Shanghai, Shanghai, China) in water (A2) and formic acid (0.1%) in acetonitrile (B2, Thermo Fisher) or ammonium formate (5 mM, Sigma Aldrich, St. Louis, MO, USA,) in water (A3) and acetonitrile (B3) was set to carry out the gradient elution of analytes at the flow rate of 0.25 mL/min. After equilibration, 2 μL of each sample was injected. A growing linear gradient of solvent B2/B3 (v/v) was applied as the following: 2% B2/B3 (0–1 min), 2%-50% B2/B3 (1–9 min), 50%-98% B2/B3 (9–12 min), 98% B2/B3 (12–13.5 min), 98%-2% B2/B3 (13.5–14 min), 2% B2 (14–20 min, positive model), 2% B3 (14–17 min, negative model).

### Mass spectrometry condition *[19]*

With the spray voltage of −2.5 kV in negative mode and 3.5 kV in positive mode, Thermo Q Exactive mass spectrometer (Thermo Fisher) was employed for executing the electrospray ionization ion-mass spectrometry (ESI-MSn) experiments. Auxiliary gas and sheath gas were set at 10 and 30 arbitrary units, respectively. The temperature of capillary was 325°C. At the mass resolution of 70,000, an analyzer scanned for full scan over a mass range of 81–1 000 m/z. Higher energy collisional dissociation (HCD) scan was carried out for the Data dependent acquisition (DDA) tandem mass spectrometry (MS/MS) experiments. 30 eV was regarded as the normalized collision energy. Some unnecessary information from MS/MS spectra was removed by dynamic exclusion.

### Determination of CSF inflammatory cytokines *[21]*

Inflammatory cytokines in CSF were determined using Enzyme-linked immunosorbent assay (ELISA). In brief, CSF samples were collected and centrifuged for 10 min at 800 g. After that, supernatants were then stored at −80°C until further analysis. Commercially available ELISA kits (R&D Systems, Minneapolis, MN, USA) were used for measuring the levels of interleukin 8 (IL-8), interleukin 6 (IL-6) and tumor necrosis factor alpha (TNF-α) in CSF in accordance with the manufacturers’ instructions.

### Statistical analysis *[22]*

The collected data was modeled by applying projection method-based multivariate data analysis. Specifically, Principal Component Analysis (PCA) was conducted for exploratory data analysis.

The differences between these two groups (normal controls and neurobrucellosis) were identified by using Variable Influence on Projection selection (VIP)-based Projection to Latent Structures Discriminant Analysis (PLS-DA). For the results from ELISA, data were exhibited as mean ± standard deviation (SD). The differences between two groups were analyzed by nonparametric test and p < 0.05 was considered as statistical significance.

### The relevant variables identification *[22]*

For identifying relevant variables describing these two groups emerging from the stability selection procedure, massbank (http://www.massbank.jp/), METLIN metabolite database (https://metlin.scripps.edu/), Human Metabolome DataBase (HMDB; http://www.hmdb.ca/), mzclound (https://www.mzcloud.org) and LipidMaps (http://www.lipidmaps.org) were applied. The fragmentation spectra (analysis in MS^E^ mode) and the mass to charge ratio (m/z) of the extracted compounds were compared to those compounds listed in these online databases. A difference of 8 ppm was established between accurate mass from mass spectrometry and exact mass from these databases for hypothetical identification aims (referred to as annotation), also including the low-intensity mass measured in positive and negative ionization mode by instrument. In addition, the indicator of the most possible structure in those discovered in these databases was achieved by assessment of the polarity and the retention time of the compounds: similar structures showed similar retention time and similar polarity in chromatographic column. Furthermore, for comparing the fragmentation spectra we noticed with those in these databases and for acquiring more data about compounds’ structure and classes, MS^E^ analysis was executed. We compared the fragmentation spectra and the retention times of standards with the ones of the emerged variables, for those compounds whose standards were available.

## Results

### Clinical findings

We aimed to find prominent CSF metabolic biomarkers using LC–MS based metabolomics. Therefore, 25 neurobrucellosis patients diagnosed clinically among patients with suspected CNS infectious were enrolled. Ten were women (41–61 years old) and 15 were men (28–62 years old). Their clinical manifestations, the results of brucellosis genus and anti-brucellosis antibodies detected in CSF samples are summarized in Table 1. Subsequently, CSF samples were collected for LC-MS measurement.

**Table 1.** Summary of research participants

	Brucell (n = 25)	Normal (n = 25)
Male	n = 15	n = 19
Female	n = 10	n = 6
Duration of disease (months)	5 (2018.04–2018.08)	9 (2018.03–2018.12)
Fever	n = 14	0
Headache	0	8
Hearing loss	1	0
Seizure	3	4
Meningeal signs	0	0
Brucella in CSF	0	0
Brucella antibodies in CSF	n = 9	0

### Metabolic profiling

**Figure 1.**
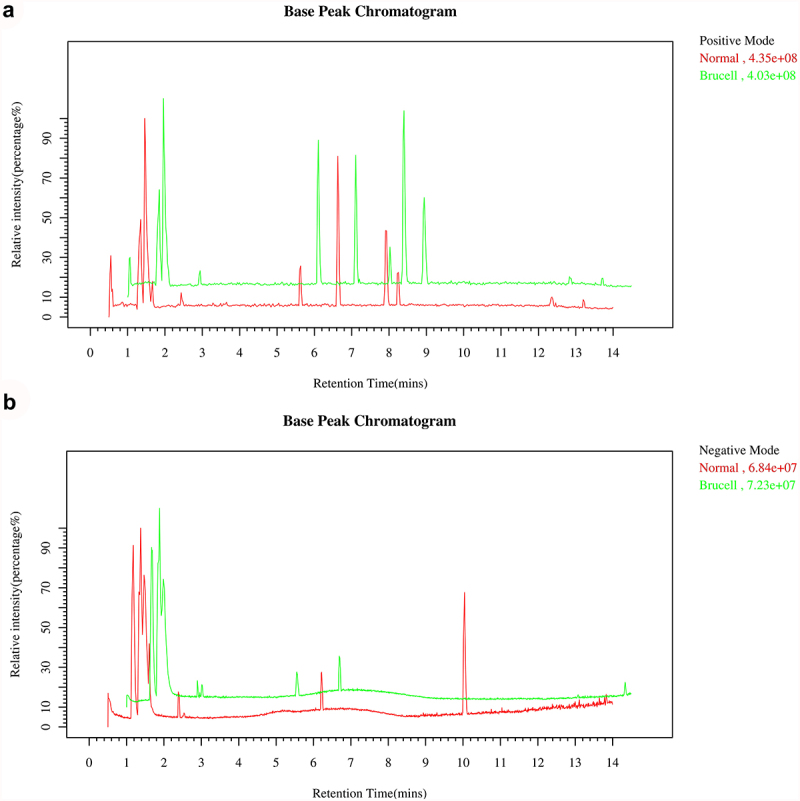
The Base peak chromatogram from Brucella cases (green) and normal controls (red) in the positive (a) and negative (b) datasets.

Base peak chromatograms of the CSF samples were applied to verify a differential ion spectrum which showed differences in metabolite contents of the two groups. The representative base peak chromatogram of CSF samples in LC–MS positive ion mode and negative ion mode were shown in Figure 1(a,b). It could be observed that due to the small particle size (1.8 μm) of the HSS T3 column packing and the high-performance of the chromatography system, small-molecule metabolites were well separated in the short elution time.

### PCA scores plots analysis

**Figure 2.**
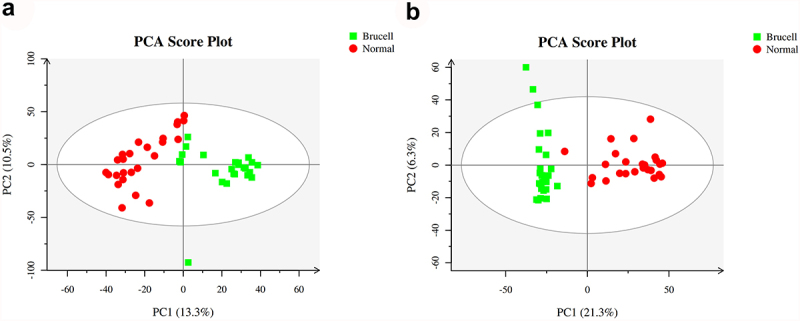
The score scatter plots of ESI^+^ mode (a) and ESI^−^ mode (b) in Principal Component Analysis (PCA) model; green square indicated Brucella cases, and red circles indicated normal group. Axes showed the percentage of variance of the first two components (PC1, PC2).

At the first stage in the data analysis, an exploratory data analysis was conducted using PCA. 23.8% and 27.6% of the total variance were explained by the first two principal components in positive and negative ion modes, respectively (Figure 2(a,b)). The data presented that on the basis of their group, these detected samples exhibited to be arranged into two main clusters.

### Partial Least Squares Discriminant Analysis (PLS-DA)

**Figure 3.**
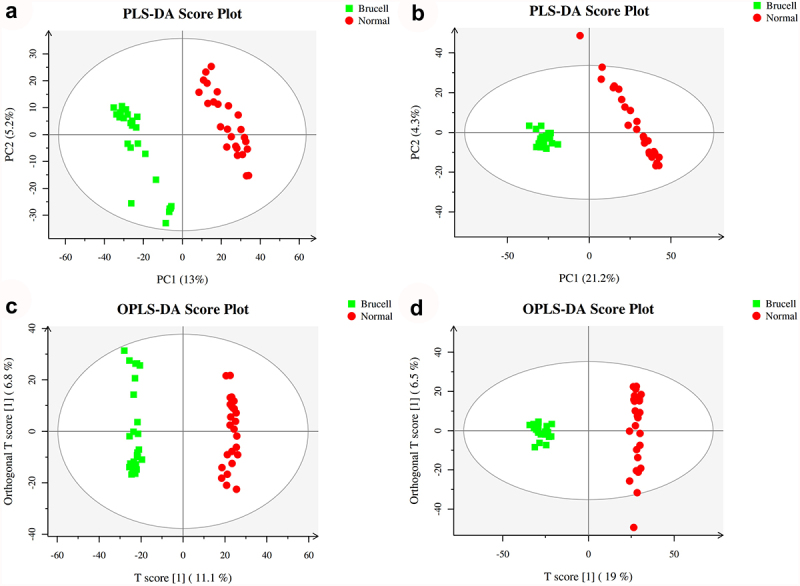
Score plots of partial least squares discriminant analysis (PLS-DA) in CSF samples of normal controls (red circles) and Brucella cases (green square) in the positive (a) and negative (b) datasets; Score scatter plots of positive (c) and negative (d) datasets in OPLS-DA model; green square indicated Brucella cases, while red circles indicated normal group. Axes represented orthogonal component and predictive component of the models.

PLS-DA is a supervised analysis method which is applied to discriminate metabolites between groups. The scores plot of PLS-DA of samples in electrospray ionization positive-ion mode (ESI^+^) and electrospray ionization negative-ion mode (ESI^−^) mode are shown in Figure 3(a,b). The outcomes showed a noticeable separation was observed between neurobrucellosis and normal controls. In a word, PLS-DA was a method that could preliminarily differentiate difference among groups. Further study according to the supervised analysis was required for studying the detailed variations correlated to neurobrucellosis.

### Orthogonal Partial Least-Squares-Discriminant Analysis (OPLS-DA)

R2X, R2Y and Q2 were the parameters for assessing the quality of the OPLS-DA model. Likewise, the score plots of OPLS-DA from normal controls and neurobrucellosis proved outstanding performance. One principal component and one orthogonal component were showed in positive ion mode with Q2 of 0.92, R2X of 0.262 and R2Y of 0.993 (Figure 3(c)). One principal component and two orthogonal components were presented in negative ion mode with Q2 of 0.933, R2X of 0.255 and R2Y of 0.991 (Figure 3(d)). In accordance with the Q2Y and R2Y parameters, the differences between normal controls and neurobrucellosis were reliably explained in OPLS-DA model. These data demonstrated that the LC–MS resulted from positive ion mode and negative ion mode had nearly identical predictability power and goodness of fit, indicating that CSF metabolites in normal controls and neurobrucellosis were remarkable different.

### Novel potential biomarker identification

**Figure 4.**
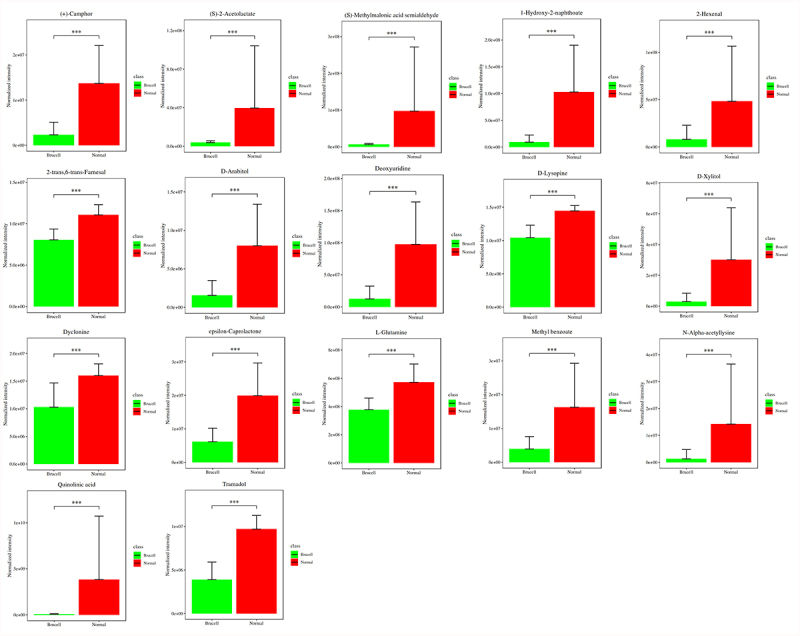
Potential biomarkers were detected using LC-MC approach in CSF samples. The figures normalized to total peak area of each metabolite were presented as mean + SD. *p < 0.05, **p < 0.01, ***p < 0.001 in comparison to normal controls.

**Figure 5.**
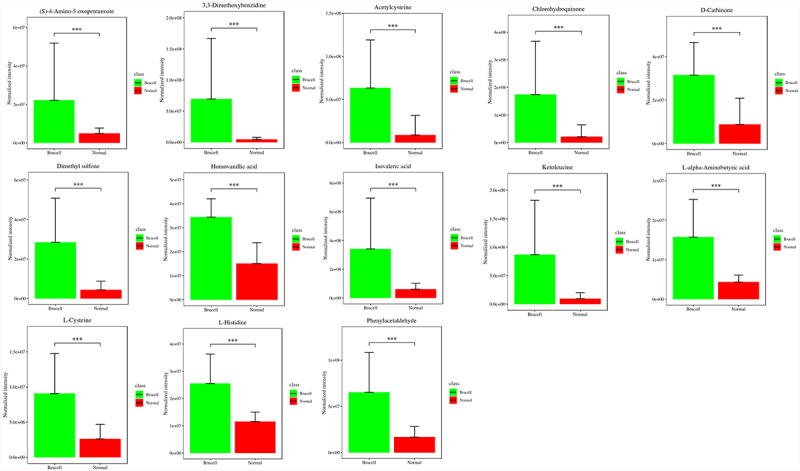
Potential biomarkers were detected using LC-MC approach in CSF samples. The figures normalized to total peak area of each metabolite were presented as mean + SD. *p < 0.05, **p < 0.01, ***p < 0.001 in comparison to normal controls.

**Figure 6.**
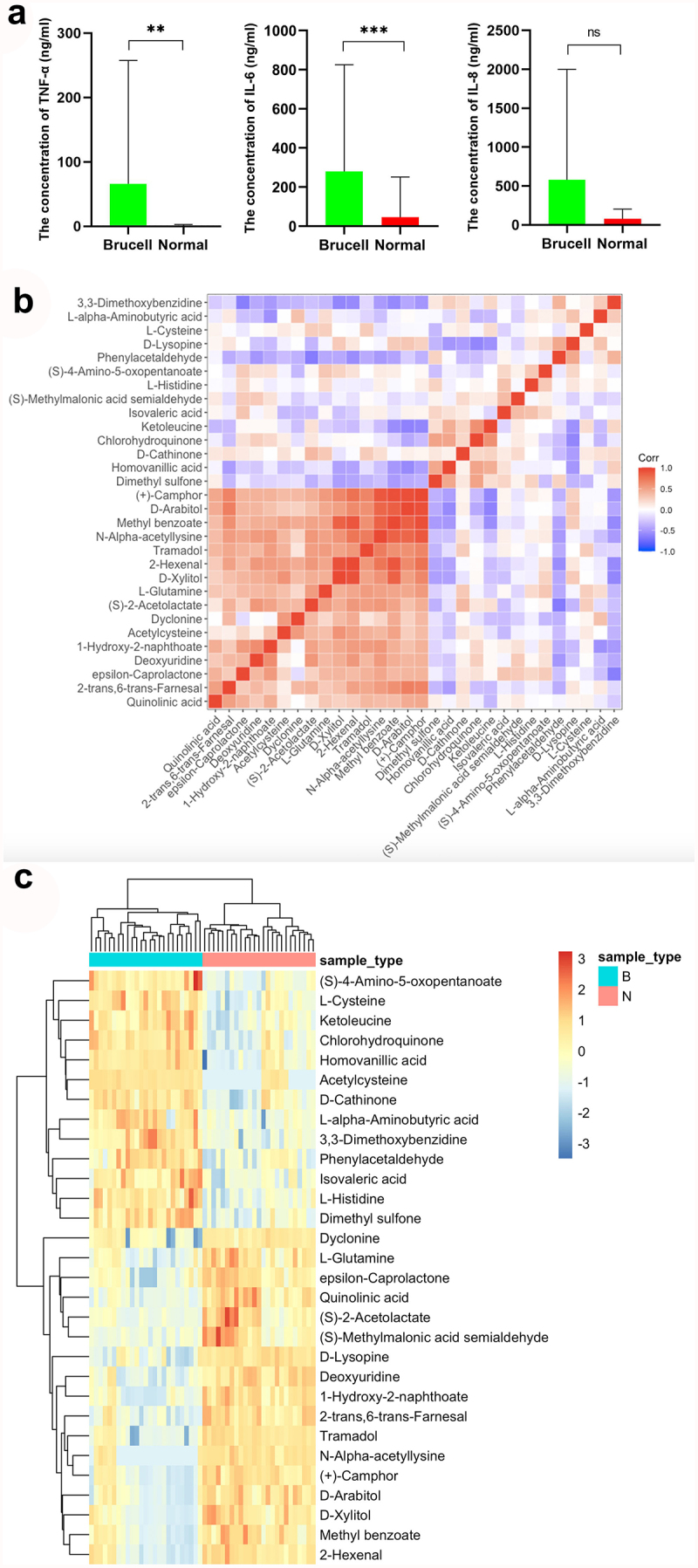
The levels of inflammatory cytokines TNF-α, IL-6 and IL-8 were measured with ELISA (a); The correlation between 30 metabolites in CSF samples (b) and their normalized heatmap (c) in neurobrucellosis cases. **p < 0.01, ***p < 0.001 in comparison to normal controls.

To discover the differences of metabolites levels, P values (P < 0.05) in t test combined with variable importance in the projection (VIP) values (VIP > 1) in OPLS-DA model were adopted. Compared to normal controls, neurobrucellosis showed a total of 155 differentiated metabolites (Table 2). Prominent potential biomarkers including 30 metabolites (Figures 4 and 5) between normal controls and neurobrucellosis samples were screened out, referred to as more capable of distinguishing neurobrucellosis from normal controls. Central nervous system (CNS) invasion by Brucella results in an inflammatory disorder in neurobrucellosis [23]. Infection with Brucella abortus elicits the production of pro-inflammatory cytokines that contribute to CNS damage [24,25]. Inflammatory cytokines TNF-α, IL-6 and IL-8 in CSF of neurobrucellosis and normal controls were also measured. The results showed that compared to that of normal controls, the levels of TNF-α and IL-6 were significantly elevated, and IL-8 level was increased but without statistical significance (Figure 6(a)), indicating the CNS involvement of the patients. Then the heatmap of the discovered latent biomarkers showed certain clustering tendencies on the basis of their relative intensity (Figure 6(c)). Several identified biomarkers, for instance 2-Hexenal and D-xylitol, were highly correlated, revealed in correlation analysis (Figure 6(b)). Hence, these metabolic alterations might reflect the CNS response to Brucella infection.

**Table 2.** Applying orthogonal principal component analysis model, differentiated metabolites were discovered between normal and Brucella cases

Metabolites	VIP	p.value	Fold Change
Tramadol	2.57	4.13E-09	0.40
3,3-Dimethoxybenzidine	1.31	4.64E-09	15.12
D-Lysopine	2.46	1.17E-08	0.72
*Quinolinic acid [32,36,60]	1.11	1.64E-08	0.02
*Deoxyuridine [55]	2.00	2.3E-08	0.13
1-Hydroxy-2-naphthoate	1.83	2.87E-08	0.09
(S)-4-Amino-5-oxopentanoate	1.16	4E-08	4.51
Dimethyl sulfone	1.82	4.46E-08	6.42
*L-Histidine [39]	2.00	5.55E-08	2.21
Acetylcysteine	1.65	7.11E-08	7.15
(+)-Camphor	2.05	7.68E-08	0.17
N-Alpha-acetyllysine	1.15	7.95E-08	0.09
*Homovanillic acid [61–63]	2.32	8.55E-08	2.28
2-trans,6-trans-Farnesal	2.32	8.55E-08	0.73
*D-Xylitol [54]	1.50	9.51E-08	0.10
Chlorohydroquinone	1.47	1.18E-07	8.09
*D-Arabitol [52]	1.90	1.62E-07	0.19
L-Cysteine	1.84	2.21E-07	3.47
epsilon-Caprolactone	2.06	3.02E-07	0.31
Ketoleucine	1.51	3.7E-07	9.03
Isovaleric acid	1.49	4.1E-07	5.52
Dyclonine	1.95	5.56E-07	0.64
D-Cathinone	1.95	6.8E-07	3.56
*L-alpha-Aminobutyric acid [64]	1.94	9.16E-07	3.61
Phenylacetaldehyde	1.86	1.12E-06	3.88
(S)-2-Acetolactate	1.12	1.12E-06	0.10
*2-Hexenal [50,51]	1.31	1.23E-06	0.17
*L-Glutamine [57,58]	2.03	1.23E-06	0.66
Methyl benzoate	1.66	1.36E-06	0.24
(S)-Methylmalonic acid semialdehyde	1.06	1.65E-06	0.07
beta-Alanine	1.95	1.81E-06	1.34
Scopoletin	2.07	1.81E-06	1.18
1-Hexadecanol	2.12	1.81E-06	2.83
Aspartame	1.85	1.81E-06	0.23
2-Aminoisobutyric acid	1.44	2.92E-06	0.18
1,2-Benzoquinone	1.61	2.92E-06	1.60
Pipecolic acid	1.95	2.92E-06	0.65
cis-4-Hydroxy-D-proline	1.91	3.21E-06	1.53
Methylimidazoleacetic acid	1.81	3.53E-06	0.37
trans-trans-Muconic acid	1.22	3.66E-06	3.72
Guanine	1.99	4.26E-06	0.69
Glutaric acid	1.88	5.12E-06	0.52
Kynurenic acid	1.42	5.12E-06	1.78
Spermidine	1.27	5.35E-06	0.32
Rimantadine	1.99	6.75E-06	1.18
Sorbitol 6-phosphate	1.47	7.39E-06	0.13
2-Oxoarginine	2.00	1.16E-05	0.38
Adenine	1.88	1.38E-05	0.66
Succinic acid	1.74	1.51E-05	0.38
Cortexolone	1.76	1.51E-05	0.45
Sphinganine	1.57	1.8E-05	0.87
Porphobilinogen	1.92	2.55E-05	1.12
5-Hydroxymethyluracil	1.40	3.29E-05	2.02
O-Acetylserine	1.68	3.29E-05	1.98
L-Fucose	1.59	3.29E-05	0.38
Myristic acid	1.62	3.9E-05	0.90
Pyrrole-2-carboxylic acid	1.27	4.24E-05	1.71
4-Hydroxy-2-oxoglutaric acid	1.73	4.24E-05	1.53
Pterin	1.53	4.24E-05	0.28
3-Methyl-L-tyrosine	1.81	4.24E-05	1.33
Gyromitrin	1.68	5.44E-05	0.67
Phosphoserine	1.19	5.44E-05	0.32
5-(2-Hydroxyethyl)-4-methylthiazole	1.10	5.55E-05	0.36
O-Toluidine	1.65	5.91E-05	3.09
L-Lysine	1.53	5.91E-05	3.70
Fomepizole	1.55	6.96E-05	0.32
3-Dehydroshikimate	1.16	6.96E-05	0.31
4-Hydroxystyrene	1.17	7.55E-05	0.46
1-Methylxanthine	1.79	7.55E-05	0.52
Indole	1.60	8.19E-05	0.92
Lumichrome	1.67	8.19E-05	0.58
Azatyrosine	1.11	8.88E-05	2.61
Diphenylamine	1.23	9.62E-05	0.88
8-Amino-7-oxononanoate	1.03	0.000113	0.79
5-Guanidino-3-methyl-2-oxopentanoate	1.33	0.000122	1.73
5-(Methylthio)-2,3-dioxopentyl phosphat	1.37	0.000122	0.32
L-Asparagine	1.38	0.000132	0.68
Dodecanoic acid	1.76	0.000195	1.51
(S)-4-Hydroxymandelate	1.18	0.000227	1.84
2-Methylserine	1.49	0.000357	0.74
Hydrogen phosphate	1.59	0.000384	1.15
5-Acetamidovalerate	1.44	0.000384	1.52
Vanillylmandelic acid	1.84	0.000445	1.17
Nicotine	1.49	0.000553	1.44
Choline sulfate	1.45	0.000553	0.56
Tartaric acid	1.13	0.000594	2.98
L-4-Hydroxyphenylglycine	1.62	0.000594	0.51
Sumatriptan	1.31	0.000789	1.98
Acetylphosphate	1.47	0.001671	2.47
2-Dehydropantoate	1.09	0.002035	0.78
Maleic acid	1.46	0.002172	0.33
Levofloxacin	1.51	0.002172	36.51
Undecanoic acid	1.22	0.003186	1.43
Indican	1.54	0.003186	2.02
Hydroxykynurenine	1.32	0.00384	1.10
L-Alanine	1.12	0.004614	1.24
Deoxyribose	1.23	0.004902	1.70
2-Pyrocatechuic acid	1.19	0.005527	0.94
Guanidinosuccinic acid	1.35	0.006223	2.26
Fraxetin	1.02	0.0066	2.76
Norepinephrine	1.58	0.010432	0.78
2-Keto-6-aminocaproate	1.26	0.013007	1.62
(R)C(S)S-Alliin	1.01	0.018887	0.66
Cyclizine	1.28	0.023199	1.11
trans-Ferulic acid	1.12	0.028342	0.90
D-Fructose	1.02	0.029771	1.27
3-Hydroxyanthranilic acid	1.05	0.031262	1.19
Hypoxanthine	1.09	0.036126	0.77
Azacitidine	1.08	0.037884	0.77
LysoPA(16:0/0:0)	1.02	0.04162	0.67
3-Hydroxyphenylacetic acid	1.09	0.043602	0.45
5-Aminopentanoic acid	1.69	9.73E-11	948,880.00
Picolinic acid	1.49	5.93E-10	31.49
Betaine	2.14	1.42E-09	0.70
Phenyl acetate	1.23	1.46E-09	19.08
Sorbitol	2.03	1.8E-09	0.76
Theophylline	1.18	1.89E-09	17.08
Galactitol	1.09	3.26E-09	57.91
Pelargonic acid	1.56	1.46E-08	0.25
L-2-Hydroxyglutaric acid	1.25	6.18E-08	15.23
(2S)-Liquiritigenin	1.41	6.89E-08	6.51
1D-Myo-inositol 1,4,5,6-tetrakisphospha	1.47	1.45E-07	2.60
Erucic acid	1.21	2.21E-07	0.38
Glucose 6-phosphate	1.60	2.45E-07	2.27
Citramalic acid	1.65	4.54E-07	3.27
Dihydrouracil	1.48	7.51E-07	4.25
Phenylethylamine	1.40	7.51E-07	0.33
17a-Estradiol	1.45	9.16E-07	2.17
5-Hydroxypentanoic acid	1.58	2.2E-06	5.02
3-Methylthiopropionic acid	1.36	2.66E-06	3.82
Maltol	1.39	3.88E-06	0.31
Folic acid	1.11	4.26E-06	0.43
L-Arginine	1.53	4.67E-06	1.41
6beta-Hydroxytestosterone	1.25	1.16E-05	3.22
6-Hydroxynicotinate	1.42	1.97E-05	0.59
6-Methylmercaptopurine	1.17	2.15E-05	3.40
Mannitol	1.38	2.15E-05	1.18
Pyruvic acid	1.39	2.55E-05	0.49
Pyroglutamic acid	1.39	4.24E-05	0.59
Equol	1.30	4.24E-05	2.91
Nicotinic acid	1.13	5.01E-05	0.24
2-Ketobutyric acid	1.11	5.44E-05	1.31
D-Ornithine	1.33	6.96E-05	1.32
Alpha-D-Glucose	1.34	6.96E-05	2.92
Dibutyl phthalate	1.29	0.000104	1.07
[8]-Shogaol	1.15	0.000132	2.60
D-Galactose	1.12	0.000167	3.29
Creatine	1.17	0.000265	1.42
1,2,3-Trihydroxybenzene	1.08	0.000357	0.48
Bovinic acid	1.08	0.000384	0.27
L-Glutamic acid	1.17	0.000735	0.51
Pseudouridine	1.14	0.001671	1.79
Uridine	1.09	0.00384	2.30
Sarcosine	1.02	0.007415	0.52
L-Lactic acid	1.00	0.009863	0.40

*: The metabolites that have been previously associated to other diseases in the literature.

(): The literatures in References list.

VIP: contribution of the metabolite to model construction; fold change: base-2 logarithm value of ratio between brucell and normal groups; p value: statistical p value.

## Discussion

Brucellosis is a weakening febrile sickness triggered by Brucella, a zoonosis of more than 500,000 new cases annually [1]. Advancement has been made over the past two decades to well learn the numerous characteristics of human brucellosis, in the meantime, great challenges remaining in monitoring and eliminating brucellosis include innovative diagnostic instruments [26,27]. In our study, 25 patients were diagnosed to neurobrucellosis with suspected infectious CNS illness clinically, with considerably different symptoms. Metabolomics measurement in CSF using LC-MS approach showed prominent potential biomarkers including 30 metabolites. To our knowledge, this is the first study to measure CSF metabolomics in neurobrucellosis patients.

Neurobrucellosis is the most morbid form in brucellosis disease [3]. However, neurobrucellosis show various clinical manifestations, and the symptoms are always atypical [2], which is strongly similar with other CNS infectious diseases, such as syphilis and tuberculosis [28,29]. Therefore, prompt and accurate diagnosis for initiating suitable therapy is essential for beneficial outcome for neurobrucellosis patients. Clinical manifestations in our 25 patients varied dramatically. Gold standard for diagnosis is the positive culture, whereas the sensitivity in routine culture measurement is low. Approximately 15% of neurobrucellosis patients has positive CSF cultures while 28% has positive blood cultures [30]. In our study, we found no patient was cultured positively. Besides, CSF culture is time-consuming for diagnosing neurobrucellosis, making prompt diagnosis a challenging mission. In comparison with routine culture test, serology is faster and more sensitive, while brucellosis seropositivity in endemic area is higher, hence it can not always recognize those previous or active infections [17].

Brain energy metabolism alters in acute inflammation and is related with the suppressed spontaneous activity, impaired cognition and delirium [31]. Elevation of CSF quinolinic acid and progression of subacute sclerosing panencephalitis indicate a pathological role of kynurenine pathway metabolism in inflammatory neuro-destruction [32]. Some clinical research and case reports have proved that metabolomics analysis is a diagnostic mean for infectious illnesses. CSF mass-spectrometric profiling shows metabolite biomarkers for CNS involvement in varicella zoster virus reactivation [33]. LC-MS method is utilized for measuring CSF metabolites in acute CNS infection [34]. In our study, the CSF samples of 25 normal controls and 25 neurobrucellosis patients were collected for metabolite detection by using LC-MS approach. The base peak chromatogram of CSF samples showed well separation of small-molecule metabolites. PCA scores plots analysis presented that the detected samples were arranged into two main clusters in accordance with their group. A distinct separation was observed between the two groups, as indicated by PLS-DA. According to the parameters R2Y and Q2Y, the differences between neurobrucellosis and normal controls could be reliably explained in OPLS-DA models.

Previous research suggests CSF metabolomics may be a fast screening measurement to improve diagnostic correctness and enhance patient prognosis [35]. The combined changes in neopterin, nitric oxide pathway and tryptophan-kynurenine pathway represent a valuable latent panel for neuroinflammation [36]. The potential of CSF metabolites has been regarded as additional diagnostic tools for enteroviral meningitis [37]. Therefore, we next further identified novel potential biomarker for neurobrucellosis. Neurobrucellosis showed a total of 155 differentiated metabolites. Prominent potential biomarkers including 30 metabolites were then selected out, regarded as more capable of distinguishing neurobrucellosis. Among them, seventeen metabolites were reduced in neurobrucellosis, including (+)-Camphor, (S)-2-Acetolactate, (S)-Methylmalonic acid semialdehyde, 1-Hydroxy-2-naphthoate, 2-Hexenal, 2-trans,6-trans-Farnesal, D-Arabitol, Deoxyuridine, D-Lysopine, D-Xylitol, Dyclonine, epsilon-Caprolactone, L-Glutamine, Methyl benzoate, N-Alpha-acetyllysine, Quinolinic acid, Tramadol. Besides, thirteen metabolites were elevated in neurobrucellosis, including (S)-4-Amino-5-oxopentanoate, 3,3-Dimethoxybenzidine, Acetylcysteine, Chlorohydroquinone, D-Cathinone, Dimethyl sulfone, Homovanillic acid, Isovaleric acid, Ketoleucine, L-alpha-Aminobutyric acid, L-Cysteine, L-Histidine, Phenylacetaldehyde. We presented the heatmaps and correlation analyses among the identified 30 potential biomarkers. Previous studies have also identified certain metabolites as markers for CNS infectious diseases. For instance, kynurenine is one of CSF biomarkers for identifying viral or bacterial CNS infections [38]. Metabonomics study finds that palmitoyl-L-carnitine, butyryl-L-carnitine, alpha-kamlolenic acid, prostaglandin E2, l-histidine and bilirubin are recognized as new prospective biomarkers for neurosyphilis [39].Twenty-five vital metabolites can be latent biomarkers in tuberculous meningitis differential diagnosis [40]. Our study demonstrates the power of metabolomics detection for search out potential metabolites in CSF as biomarkers for neurobrucellosis.

Previous literatures have revealed that acetyl-cysteine (NAC) is a precursor of L-cysteine and is recognized to effectively cross blood-brain barrier [41]. Cysteine plays an essential role in redox homeostasis, being a component of glutathione (GSH) [42]. Importantly, GSH is the most plentiful non-protein thiol and a critical cellular antioxidant, protecting brain cells against oxidative stress and maintaining redox homeostasis [43,44]. Mitochondrial proteolytic pathways, phospholipid metabolism and redox metabolism are discovered to be the key and latent free radical sources [45]. A growing consensus manifests that mitochondrial GSH is crucial for the defense of cellular antioxidant [46]. ELISA showed that inflammatory cytokines TNF-α, IL-6 and IL-8 in CSF of neurobrucellosis were obviously elevated. We speculated that abnormal accumulation of L-cysteine and acetylcysteine in neurobrucellosis CSF may be due to the inflammation-induced mitochondrial dysfunction and subsequent neurological damage. Further studies need to be performed for a better stratification and understanding.

(S)-2-acetolactate can be catalyzed and converted into (R)-2,3-dihydroxy-isovalerate by Ketol-acid reductoisomerase (KARI), one of important enzymes in the BCAA pathway [47]. 1-hydroxy-2-naphthoate has been proposed to be an intermediate product in the degradation pathway of phenanthrene [48,49]. A mixture containing trans-2-hexenal attenuates fear, anxiety and stress responses in rodents [50,51]. D-arabitol and ribitol are metabolic end-products in human [52]. The major polyols include ribitol and xylitol [53], and both of them are sugar metabolites [53]. Xylitol increases cerebral blood flow in hypothalamus [54]. Deoxyuridine can be catalyzed and phosphorylated to the corresponding uracil by thymidine phosphorylase [55]. Deoxyuridine nucleotides are the precursors for de novo synthesis of the deoxythymidine nucleotides [55]. L-glutamine is the most abundant amino acid in CSF and a precursor for neurotransmitters of L-glutamate and γ-aminobutyric acid [56]. Patients with probable Alzheimer’s Disease show increased glutamine level in CSF [57]. Disrupted glutamate-glutamine cycle in CSF metabolites associated with tuberculous meningitis has been identified [58]. Quinolinic acid is a metabolite of tryptophan degradation obtained through kynurenine pathway, which can be detected in both brain and CSF [59] and has an excitotoxic effect for neurons [32]. HIV-positive participants have higher quinolinic acid in CSF [60], while there is a statistical increase of quinolinic acid in encephalitis patients [36].

Dopamine can target different receptors and is then degraded to its major metabolite homovanillic acid [61]. Homovanillic acid has been identified as a biomarker for dementia [62]. Patients with Parkinson’s disease manifesting dyskinesia are featured by the higher ratio of homovanillic acid/dopamine [63]. Alpha-aminobutyric acid is elevated in patients with Alzheimer’s disease in CSF [64]. The entry of L-Histidine in brain is correlated with histamine homeostasis [65]. One of the most important metabolites of L-Histidine in the brain is histamine, a neurotransmitter [65]. Brain histamine is changed significantly in several neurological diseases [66]. However, the potential effects of these 30 metabolites on neurobrucellosis have been scarcely studied thus far, which require further investigation.

In this study, several metabolites were changed significantly at higher fold, for example 5-aminopentanoic acid and galactitol in neurobrucellosis group. 5-aminovaleric acid participates in modulating glutamine-glutamate-gamma-aminobutyric acid (GABA) metabolic pathway [67]. 5-aminovaleric acid is an antagonist to GABAb receptors, while blocks the anticonvulsant properties of GABAb agonists [68]. Fatal cerebral edema may be related to the accumulation of galactitol, which can be synthesized by galactose [69,70]. Galactitol accumulation may play a role in the postnatal CNS dysfunction [71]. Motor nerve conduction velocity is found to be reduced, which is associated with galactitol accumulation in peripheral nerves [72]. Nevertheless, studies of 5-aminopentanoic acid or galactitol on neurobrucellosis are still limited and therefore needed further exploration.

As for the advantages of LC-MS approach, LC-MS represents a simple, rapid, robust and reliable approach for measuring metabolome in test samples [73]. LC-MS with data-independent acquisition becomes a powerful technology for metabolomics because of its highly quantitative accuracy [74]. Targeted metabolomics based on LC-MS/MS has the advantages of being quantitative, reproducible and more sensitive [75]. On the other hand, limitations in data acquisition speed, cellular temporal-specific expression alterations and wide-range of metabolites concentrations confound the understanding of metabolite perturbations [10]. As we have described, LC-MS-based metabolomics analysis has been applied in studying neurological disorders [12,13,16,34]. The use of LC-MS approach for detecting CSF metabolites can be a prospective candidate for neurobrucellosis diagnosis and prognosis assessment, while further study is necessary to develop this approach in neurobrucellosis.

## Conclusions

To sum up, novel metabolites with up/downregulated expressions can be discovered in patients with neurobrucellosis using LC-MS-based CSF metabolomics. Although this study is limited due to population size and disease heterogeneity, the presence of different metabolites provides useful information regarding potential biomarkers for neurobrucellosis.

## Data Availability

All data generated or analyzed during this study are included in this published article.
